# The Invisible Barriers to Sustainable Surgical Practice

**DOI:** 10.1002/wjs.70244

**Published:** 2026-01-23

**Authors:** Mina Sarofim

**Affiliations:** ^1^ School of Medicine University of New South Wales Australia Sydney New South Wales Australia; ^2^ School of Medicine University of Sydney Australia Camperdown New South Wales Australia

In recent years, increasing attention has been directed toward improving the environmental sustainability of surgical practice, particularly through the reduction of reliance on single‐use disposable medical devices. This movement is underpinned by compelling environmental data and bolstered by growing awareness among clinicians, policymakers, and professional societies [1, 2]. Yet despite the availability of safe, effective reprocessing technologies, and growing institutional support for sustainability initiatives, actual behavioral change within operating theaters remains limited and uneven. A critical and often underappreciated obstacle lies not in the domain of technology or policy but in psychology and culture.

Decades of cognitive science research reveals that risk perception is frequently decoupled from objective hazard. Nowhere is this misalignment more consequential than in clinical decision‐making, where deeply embedded heuristics shape routine practice. One such heuristic, known as contagion bias, refers to the instinctive aversion to objects perceived as contaminated, regardless of actual sterility or risk [3]. Despite rigorous evidence demonstrating the equivalence, or even superiority, of reprocessed devices in safety and performance, many clinicians implicitly equate disposability with sterility and quality. The psychological shorthand of “new equals clean” and “used equals risk” remains stubbornly embedded in the clinician's decision matrix.

Compounding this is the phenomenon of status quo bias: the tendency to prefer established practices simply because they are familiar. In healthcare settings, where time pressures, medicolegal considerations, and institutional dogma dominate, such conservatism is hardly surprising. Once disposability became normalized (fueled in part by industry marketing and the pursuit of streamlined logistics), it was rapidly embedded as the unchallenged standard of care [4]. Their ubiquity generates a circular logic: disposables are assumed to be superior because they are prevalent, and they remain prevalent because they are assumed to be superior.

The fundamental structure of surgical departments further amplifies resistance to change. Hierarchies in clinical teams, while essential for certain aspects of care delivery, can also entrench outdated norms. Behavioral research consistently shows that clinical behavior is highly influenced by senior figures within a team, meaning that without visible leadership endorsing sustainable device use, old habits are unlikely to die [5]. Patient expectations introduce an additional layer of complexity. In many settings, patients equate disposability with technological sophistication and modernity. There may be institutional reluctance to adopt or disclose the use of reprocessed devices out of fear that it will be perceived as a cost‐cutting measure rather than an evidence‐based, environmentally responsible choice. This perception gap further disincentivizes change and makes it politically and reputationally easier to maintain the status quo [6].

Addressing these intertwined psychological and cultural barriers demands more than regulatory endorsement or availability of alternative products. Interventions grounded in behavioral economics offer promising avenues. One such approach is an “opt‐out” system where reusable instruments are presented as the standard, unless actively declined, which has been shown to alter clinician behavior in other health care domains provoking resistance [7]. Such strategies leverage cognitive shortcuts rather than attempting to bulldoze them. Equally important is transparency. Public reporting of both the environmental and financial implications of disposable versus reusable devices may help calibrate sustainability with notions of clinical excellence and fiduciary responsibility. Reframing is essential if sustainability is to be internalized as a dimension of quality care rather than a marginal or optional concern.

Perhaps most crucially, professional leadership must rise to meet this challenge. Cultural transformation requires prominent clinicians to champion sustainable practices not only in the operating theater decision‐making but also in public discourse, clinical teaching, and policy development. Psychological barriers begin to dissolve if sustainability is the new pillar of surgical excellence, alongside safety, innovation, and patient care.

If we fail to challenge the psychological inertia that sustains the throwaway paradigm in surgery, we risk undermining the very future we are training the next generation to inherit. There is little value in perfecting surgical technique or advancing innovation if the planet that sustains our health systems and our patients cannot endure.

## Author Contributions

**Mina Sarofim:** conceptualization, investigation, writing – original draft.

## Funding

The author has nothing to report.

## Conflicts of Interest

The author declares no conflicts of interest.

## Data Availability

Data sharing not applicable to this article as no datasets were generated or analyzed during the current study.
